# Regulatory Mechanisms of Mitochondrial Function and Cardiac Aging

**DOI:** 10.3390/ijms21041359

**Published:** 2020-02-18

**Authors:** Ruizhu Lin, Risto Kerkelä

**Affiliations:** 1 Research Unit of Biomedicine, Department of Pharmacology and Toxicology, University of Oulu, FI-90014 Oulu, Finland; Ruizhu.Lin@oulu.fi; 2 Medical Research Centre Oulu, Oulu University Hospital and University of Oulu, FI-90014 Oulu, Finland; 3 Biocenter Oulu, University of Oulu, FI-90014 Oulu, Finland

**Keywords:** cardiovascular, senescence, aging, non-coding RNA

## Abstract

Aging is a major risk factor for cardiovascular diseases (CVDs), the major cause of death worldwide. Cardiac myocytes, which hold the most abundant mitochondrial population, are terminally differentiated cells with diminished regenerative capacity in the adult. Cardiomyocyte mitochondrial dysfunction is a characteristic feature of the aging heart and one out of the nine features of cellular aging. Aging and cardiac pathologies are also associated with increased senescence in the heart. However, the cause and consequences of cardiac senescence during aging or in cardiac pathologies are mostly unrecognized. Further, despite recent advancement in anti-senescence therapy, the targeted cell type and the effect on cardiac structure and function have been largely overlooked. The unique cellular composition of the heart, and especially the functional properties of cardiomyocytes, need to be considered when designing therapeutics to target cardiac aging. Here we review recent findings regarding key factors regulating cell senescence, mitochondrial health as well as cardiomyocyte rejuvenation.

## 1. Introduction

Aging is associated with progressive functional deterioration at both organ and cellular level [1]. Aging increases frailty with decreased organ/cell tolerance to stresses and serves as a primary risk factor for cardiovascular diseases (CVD) [1,2,3]. Aging-associated cardiac remodeling is characterized by increased collagen deposition, cardiac hypertrophy and stiffness of aorta as well as endothelial dysfunction [1,3,4,5]. These functional and structural changes predispose to myocardial ischemia, cardiac arrhythmias and sudden cardiac death [4,6]. The aging heart has increased oxygen and energy demand due to increased afterload and hypertrophy of cardiomyocytes (CMs), while the compromised passive relaxation due to fibrosis and abnormal Ca^2+^ handling decreases the coronary artery perfusion during diastole reducing the oxygen and energy supply. The adequate delivery of oxygen and nutrients energy is especially crucial to the cardiomyocytes due to their continuous contraction through lifespan, but with only negligible proliferative capability.

The turnover rate of human CMs is ≤1%/year and declines with aging [7], which renders the heart unable to dilute damaged cardiomyocyte organelle. On the other hand, to meet the high levels of energy demand, it holds the highest abundance of mitochondria that intrinsically serve as a major source for reactive oxygen species (ROS). Due to high energy demand of cardiomyocytes, they are particularly vulnerable to mitochondrial alterations, and the dysfunctional mitochondria results in insufficient ATP supply. An increase in mitochondrial ROS production in the cardiomyocytes has been observed during aging [8] and the CMs appear more vulnerable to mitochondrial defects and ROS damage compared to other resident cardiac cells. Typical features of mitochondrial defects, such as swelling and loss of cristae, are also observed in aged heart [1,5], which contributes to the increased generation of ROS and increased cardiomyocyte damage [8,9].

Cell senescence, first reported by Hayflick et al. [10], is characterized by irreversible arrest of cell proliferation [9,11]. With the increased understanding of the features of senescence, it is typically classified in two categories [9,11]: (1) replicative senescence that occurs during natural aging and is induced in part by the erosion of telomerase due to cell cycle progression, and (2) stress-induced premature senescence (SIPS), seen in response to cytotoxic stresses such as anti-cancer therapy [12]. While both replicative senescence and SIPS likely contribute to cardiac aging, SIPS probably pertains a more critical role in driving CM aging as the CMs have little capacity for cell cycle but higher mitochondrial mass rendering them susceptible to mitochondrial dysfunction and increased ROS [5]. 

Senescent cells accumulated during aging typically feature resistance to death, suggesting they are bypassed by aged immune system [12,13]. Thus, most anti-aging drugs are mainly targeted to selectively eliminate cells that are already senescent. Among cardiac resident cells, cardiac endothelial cells and cardiac fibroblasts are capable of renewal in adulthood [14], and thereby provides an opportunity to revive their function by deleting the senescent population. The cardiomyocytes have two features distinguishing them from other resident cardiac cells: (1) low proliferative ability and (2) high abundance of mitochondria. The former raises questions whether the cardiomyocyte population can be revived after clearing senescent CMs and if such a strategy is too simplified for CMs. Indeed, most published data lack documentation on what is the cardiac senescent cell population that are removed by senolytics and what are the cells types rejuvenated. On the other hand, given the abundant number of mitochondria in cardiomyocytes and mitochondrial dysfunction representing a hallmark of cellular senescence [1], mitochondrial dysfunction in cardiomyocytes represents a central culprit for driving cardiac aging. This is exemplified in a study with targeted overexpression of mitochondria-targeted catalase that ameliorates cardiac aging (reduces cardiac hypertrophy and fibrosis) and prolongs lifespan [15]. Notably, differently from mitochondria-targeted catalase, most transgenic mice overexpressing antioxidant enzymes fail to increase longevity [16] emphasizing the critical role of mitochondrial health in protecting from cardiac aging. Taken together, it is conceivable that tackling the issue of mitochondrial dysfunction as well as replacement of aged CMs population with healthy cell population represent two pivotal hubs for management of aging –related cardiac diseases. 

MicroRNAs (miRNAs) are short non-coding RNAs, generally about 22 nucleotides [17]. By complementary binding to their target genes 3′untranslated region, one miRNA can regulate several genes at the same time, thus serving as crucial post-translational modifier of a variety of biological functions [17,18]. In the heart, emerging evidence indicates a crucial role of miRNAs in cardiac pathologies as well as development. The multi-targets of each miRNA and their short sequence enable miRNAs to relatively easily transfer between the cells and organs; the epigenetic nature of miRNAs also allow them to rapidly respond to environment change. Further, given the considerable difference in miRNA expression during aging, the function of miRNAs in cardiac aging is likely of importance and warrants detailed investigation and annotation. 

**Figure 1:**
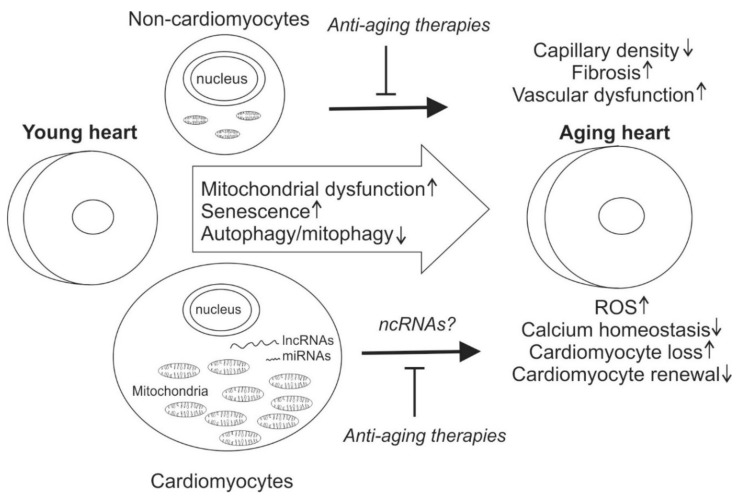
Molecular and cellular events contributing to cardiac aging. Heart consists >70% cardiomyocytes in terms of volume, whereas the turnover rate of cardiomyocytes is low. A high number of mitochondria in the cardiomyocytes serves as a major source for reactive oxygen species (ROS). Cardiac aging features abnormal calcium homeostasis, loss of cardiomyocytes, senescence and reduced cardiomyocyte renewal. Aging in non-cardiomyocytes leads to reduced capillary density, cardiac fibrosis and vascular dysfunction. Mitochondrial dysfunction, accumulation of senescent cells and impaired autophagy/mitophagy are typical cellular features of cardiac aging. Current anti-aging therapies are mainly aimed at removing senescent cells, but the targeted cell composition and the effect on cardiac function is largely unknown. MicroRNAs (miRNAs) and long non-coding RNAs (lncRNAs) play critical roles in regulating cardiomyocyte function and there is increasing evidence concerning their role in regulating cardiac aging.

Long non-coding RNAs (lncRNAs) and are defined as being more than 200 nucleotides and currently contain 9640 lncRNAs loci, representing 15,512 transcripts [19]. They have been shown to play an important role in diverse cellular processes by regulating the gene expression at transcriptional or post-transcriptional levels [20]. However, only a small fraction have been found to be present at levels high enough to suggest that they have a biological function. Contrary to the term, some of the lncRNAs encode for small peptides. Here we review recent data on factors regulating cardiac aging with focus on non-coding RNAs and their role in regulating mitochondrial function (Figure 1).

## 2. Multi-Steps of Senescence

The definition of cellular senescence encodes two key aspects: loss of cell proliferation and irreversibility [9]. Indeed, mere exit from cell cycle is defined as quiescence [11], while only the permanent duration sufficiently renders the cell as senescent. Senescence is stress-induced, and it appears that there is potential to slow or halt the progression of senescence, since if the underlying stress is transient, senescence usually does not occur [21]. Although the senescence can be classified as replicative senescence and SIPS, in essence, it is not the course of time but rather the concurrent presence of stressors acquired, or exposed to, during the lifespan that induces and sustains senescence. Unlike apoptosis, which removes cells, the senescent cells accumulate in tissues during natural aging. While there are several features of cellular senescence, the in vivo recognition of senescent cells is a challenge and their detection is currently based on detection of SA-β-gal and p16 [12,21]. Interestingly, cellular senescence is mediated by two main pathways: p53/p21 and p16 [1,11,12,22]. P53/p21 activation in response to stimuli occurs usually earlier and induces loss of cell proliferation, but not necessarily ending up with senescence or death. The induction of p16 occurring secondary to p53/p21 activation leads to a full irreversible senescence [12,23]. 

Indeed, in a model of p53 overexpression induced-cell senescence, which is accompanied by increased ROS, Akt and p21 pathways were shown to separately control different aspects of senescence [24]. Akt inhibitor treatment abolishes the increase in ROS but fails to reverse the proliferative arrest, whereas p21 inhibition can restore cell proliferation without influence on ROS production. In addition, the Akt activation is noted prior to the onset of senescence, and abrogation of Akt signal during early phase of p53 overexpression is more effective to prevent senescence phenotype [24]. In vitro, overexpressing oncogenic *ras*-induced cell senescence is featured as mitochondrial dysfunction, including elevated mitochondrial mass and mitochondrial ROS, all of which appear prior to cell evolved into senescence [25]. 

Study of irradiation or telomere dysfunction -induced senescence unveils a feedback loop between ROS generation and DNA damage, which sustains a dynamic short-lived DNA damage to enable a deeply locked cell senescence [26]. Importantly, although a minimum frequency of DDR is necessary to initiate growth arrest, it does not permit an irreversibility of cell cycle exit. Instead, (1) long-term cell-autonomous replenishing signals are triggered within 1–2 days, and the full activation of signaling is not established until several days after initial damage; (2) the irreversibility of senescent growth arrest is established on an actively-maintained equilibrium between damage and repair, which indicates that interrupting the positive feedback at late time point is also sufficient to mitigate the senescent phenotype [26]. Moreover, using a temporal combination of antioxidant, mitochondrial uncoupler and lysosomal enzyme activity enhancing agents, Haoran et al. showed that the mitochondrial dysfunction and elevated ROS occur earlier than the lysosomal dysfunction, while lysosomal dysfunction more directly leads to defective autophagy and senescence [27]. 

Taken together, senescence is not a one-step process, but instead, it is regulated by multiple signaling mechanisms that gradually promote the transition to an irreversible senescence status, all of which offers multiple phases and targets to intervention. As to adult cardiomyocytes, which only have limited proliferative capacity [28] and retain a fairly constant number through lifespan [14], protecting cardiomyocytes from injuries that contribute to senescent phenotype is of major importance and provides a viable strategy to prevent or delay cardiomyocytes senescence.

## 3. Anti-Aging Therapies

One of senescent cells traits is resistance to apoptosis, which plays a key role in senescent cell retention and accumulation [9,11]. Much of recent focus in research for therapies to reverse or mitigate aging has focused on selective clearance of senescence cells, termed as senolytics. Global depletion of naturally accumulated senescent cells during aging has shown beneficial effects [12,29], and, in general, induction of apoptosis selectively in senescent cells appears to provide benefit against aging-associated diseases. In the following section, we will review recent findings concerning anti-aging therapies to against aging and cell senescence, then focusing on cardiac senescence.

Senescent cells have been shown to be resistant to both extrinsic and intrinsic apoptosis. One mechanism of senescent cells survival is offered by upregulation of anti-apoptotic proteins BCL2L2 and BCL-XL, and targeting those factors by small interfering RNA (siRNA) or by a small-molecule ABT-737 triggers senescent cells apoptosis [30]. ABT737 has also shown efficacy in removing p14 -induced senescent cells in the skin, and, importantly, the elimination was accompanied with hair-follicle stem cell re-entry into cell cycle, suggesting an improvement of tissue renewal and fitness [30]. Likewise, another anti-apoptotic molecule ABT263, an inhibitor of BCL-2 and BCL-xL, has shown promising senolytic efficacy inducing apoptosis and eradicating senescent cells in culture [31]. Further, ABT263 showed efficacy in ablating senescent cells (senescent marker p16 labeled) in models of naturally aged mice or mice subjected irradiation injury [31]. In the latter model, ABT263 also reversed irradiation injury -induced aging by rejuvenating senescent hematopoietic cells and muscle stem cells. Recently, two studies reported that cardiac glycosides targeting Na^+^/K^+^ATPase pump have broad-spectrum senolytic effect in oncogene-induced senescence [32,33]; and the senolytic effect involves induction of Bcl-2 family protein NOXA [33].

High-throughput sequencing of normal or senescent preadipocytes (induced by irradiation) showed an increase of negative apoptosis and anti-apoptotic signaling during cell senescence [34]. This pro-survival program was centered on key nodes, including ephrins (EFNB1 or 3), p21 (CDKN1A), PI3Kδ (PI3KCD), BCL-xL and plasminogen-activated inhibitor (PAI)-2(SERPINB2), and knockdown of each of respective genes by using siRNA specifically removes senescent cells [34]. Interestingly, by targeting those same factors with tyrosine kinases inhibitor dasatinib (D) and quercetin (Q), a flavonol that represses kinases including PI3K and serpines, selectively eliminated aged cells in vitro [34]. Further, the combination of D+Q was able to prolong healthspan, ameliorate age-associated symptoms in progeroid mice model, and boost exercise capacity in irradiation injured limb [34]. Worth noting, combined administration of D+Q in 24-month-old mice, with a single dose improved cardiac function and carotid vascular function, as assessed by increased LV ejection fraction and enhanced vascular relaxation in response to nitroprusside [34]. Moreover, in bleomycin-induced lung fibrosis model, depletion of senescent fibroblasts by D+Q enhances pulmonary function and exercise capacity [35]. 

In subsequent studies, D+Q has been shown to elicit beneficial effects on cardiac regenerative capacity, frailty, osteoporosis, muscle weakness and endurance, and to extend lifespan [36,37,38]. In studies in human, D+Q alleviated physical dysfunction in patients with idiopathic pulmonary fibrosis and in subjects with diabetic kidney disease, D+Q reduced senescence cell burden in the adipose tissue [39,40].

## 4. Cardiac Senescence

The senescent cells are known to secrete numerous cytokines and growth factors known as senescence-associated secretory profile (SASP). In the heart, the pro-inflammatory environment in activates resident cardiac fibroblasts inducing their proliferation and excess production of extracellular matrix (ECM) proteins, including collagens. Premature senescence has been observed in atrial fibrillation (AF) patients with increased level of p16 and area of SA-β-gal staining in the left atrias, which positively correlated with fibrosis [41]. Interestingly, Western blot analysis also reveals an elevated expression of collagens and TGF-β in AF patients, along with increased atrial fibrosis and with a further increase in persistent AF compared to paroxysmal AF group [41]. Study of human patients with idiopathic cardiomyopathy reveals that senescent p16 and SA-β-gal expression positively associates with fibrosis [42]. Further, in this study, increased senescence is also found in hearts of mice subjected to pressure overloaded by thoracic aortic constriction (TAC) and in cardiomyocyte-specific β1-adrenoceptor transgenic mouse model, and inhibition of senescence by genetic silencing of both p53 and p16 in mice exacerbates TAC-induced fibrosis and cardiac dysfunction. In contrast, activation of senescence by AAV9-mediated overexpression of CCN1 ameliorates TAC-induced fibrosis and improves cardiac function [42]. Similarly, increased levels of senescent makers p16 and SA-β-gal^+^ cells are observed in ischemic human myocardial samples as well as in cardiac samples of infarcted mice [43]. In post-infarcted mice, a release of SASP factors, including CCN1, MCP1, IL-1α and TNFα, occurs in myocardium, and is mediated by GATA4: in vivo, short-hairpin RNA (shRNA) based knockdown of GATA4 inhibits SASP, and exacerbates cardiac dysfunction as well as fibrosis post-MI [43]. Instead, injection of recombinant CCN1 in GATA4 knockdown mice improves cardiac function [43]. Overall, it appears that the presence of cardiac senescence in cardiac pathologies does not contribute to, but rather limits disease progression.

Interestingly, there is a difference in incidence of aging-related cardiac diseases between men and women. In the Western world, men develop ischemic heart disease 7–10 years earlier than women [44]. On the other hand, acute coronary syndrome is more common in elderly women (>75 years of age) and hypertension is also more common in women in aging population [44,45]. Heart failure occurs in older age in women than men, whereas concentric remodeling and diastolic dysfunction, hallmarks of heart failure with preserved ejection fraction (HFpEF), are more common in women [46,47]. The molecular mechanisms underlying these structural differences is not clear, but data from experimental models indicates that estrogen deficiency results in activation of RAS, increased cardiomyocyte hypertrophy and increased cardiac fibrosis [48]. Arrhythmias have been found to increase with aging and are a major risk factor for sudden cardiac death (SCD). While ischemia is the major cause of SCD [49], women are more likely to have a nonischemic cause of SCD and female subjects with SCD are older than men [50].

There is an increasing body of evidence showing that long noncoding RNAs (lncRNAs) and micro RNAs (miRNAs) are involved in cardiovascular aging. LncRNAs act as key regulators of the central aspects of cardiovascular aging (i.e., cardiac hypertrophy, fibrosis, and endothelial/vascular function), and their role in cardiac biology has been recently reviewed [51,52,53,54,55]. A number of miRNAs have been associated with regulation of senescence in various tissues, including the heart [56,57]. For example, miR-34a, which promotes cell senescence in various conditions [58,59,60], is elevated in aged mice and correlated with aging in humans [61]. Targeting miR-34a by antisense oligonucleotides in mice not only mitigated age-related cardiac cell death and functional deterioration, but also ameliorated MI-induced cardiac cell death and scar area, improved capillary density at MI border zone and alleviated post-MI cardiac dysfunction [61]. One direct target of miR-34a is Phosphatase-1 NUclear Targeting Subunit (PNUTS). Both aging and MI -related increase in miR-34a were associated with decreased PNUTS expression, and AAV-mediated overexpression of PNUTS partially reduced markers of DNA damage, attenuated telomere shortening and alleviated LV dysfunction after MI [61].

There are a number of reports on miRNAs that regulate accumulation of extracellular matrix in the heart during aging or in response to injury, such as miR-125b, miR-29, miR-21, miR-18/19 [62,63,64,65,66]. MiR-125b has been shown to play an essential role in regulating cardiac fibrosis partially via silencing multiple anti-fibrotic elements, including p53 and apelin. In vivo, miR-125b increases during angiotensin II (Ang II) infusion, and targeting miR-125b attenuates Ang II -induced cardiac fibrosis [63]. By directly targeting a plethora of fibrotic genes, miR-29 modulates cardiac fibrosis, knockdown of which at basal level enhanced collagen expression in vitro and in vivo [66]. In response to stretch, cardiomyocyte-derived miR-378 represses cardiac fibrosis in an extracellular vesicles dependent fashion in fibroblast partially via targeting MKK6 to inhibit p38 signaling [67]. MiRNA Let-7i, a member of Let-7 family that account for about 14% of all miRNAs in murine hearts [68], negatively regulates myocardial fibrosis partially by directly targeting IL-6 and collagens in fibroblasts, and restoration of which attenuated Ang II-induced cardiac fibrosis and inflammation [69].

Currently, very little is known concerning the mechanisms regulating cardiac fibroblast senescence. Aging is associated with increased plasma levels of pro-fibrotic factors, including osteopontin (OPN) and TGF-β, derived from visceral adipose tissue (VAT) [70]. Depletion of VAT in old mice or OPN knockout mice has been shown to mitigate age-related decline in cardiac function and cardiac fibrosis [70]. Intriguingly, co-staining of senescent marker p16 and fibroblast marker vimentin in VAT-removed or OPN-deficient mice showed selective induction of cardiac fibroblast senescence. Furthermore, treatment of cardiac fibroblasts with plasma from OPN^-/-^ mice plasma augments cardiac fibroblast senescence that is associated with decreased cell viability and proliferation [70]. Thus, it would appear that selective induction of cardiac fibroblast senescence provides protection from aging-associated cardiac disease and deterioration of cardiac function. Taken together, the evidence of cardiac senescence involved in various cardiac pathologies is ever-growing. Despite the evidence suggesting that senescent cell ablation provides protection against aging-associated dysfunction in other organs, it appears that the role of senescence in cardiac pathologies is at least a matter of debate, and might even play beneficial role. Further, it is not clear to what extent the contractile performance of senescent cardiomyocytes contributes to cardiac contractile function. There is also insufficient evidence of the role of other cardiac cells, e.g., cardiac fibroblasts, endothelial cells and immune cells in cardiac aging. Given the well-accepted documentation of cardiomyocyte death/loss in the aged myocardium [1,61,71] and the lack of evidence of cardiac aging at cellular and molecular level, it can be debated whether killing senescent cells, especially senescent cardiomyocytes, is an effective strategy in restoring aging-related decline in cardiac function. That is at least until there is a feasible strategy to replenish the cardiomyocyte population. 

## 5. Mitochondria in the Aging Heart

The abundancy of mitochondria in cardiomyocytes accompanied by the usage of highly oxygen-dependent fatty acids as fuel source are vital for normal cardiomyocyte contractile function, but predispose the cardiomyocytes to a higher risk for ROS damage and mitochondrial dysfunction [1,5,72]. During aging, the activity of mitochondrial complexes III and IV as well as mitochondrial content of cardiomyocytes decreases [8]. The aged heart thus has decreased capacity for fatty acid usage and a shift to increased usage of glucose in energy metabolism occurs [73]. The similar metabolic shift is also typical in heart failure and is accompanied by changes in metabolic gene expression profile resembling that in the fetal heart with relative anaerobic environment [74,75,76,77]. In addition to decreased oxidative phosphorylation capacity, the shift in energy metabolism in the aged heart may stem from an attempt to minimize usage of oxygen, owing to decreased capillary density in the myocardium. 

In the healthy heart, mitochondrial quality is controlled by a dynamic balance between elimination of damaged parts by mitochondrial autophagy (mitophagy) and mitochondrial biogenesis. Mitochondrial dysfunction seems indispensably intertwined with aging: mitochondrial function and quality decreases in all tissues during aging [72], mitochondrial DNA (mtDNA) mutations occur across species and genetically modified mice with increased mtDNA mutations shortens lifespan and rapidly acquires aging phenotype [78], mitochondrial dysfunction induced by mitochondrial DNA double strand breaks (mtDSB) activates a premature senescence phenotype in mice [79], in vitro mitochondrial dysfunction promotes senescence in both post-mitotic and mitotic cells [80], and finally, restoring mitochondrial function rescues senescence and preserves cardiac function [81]. Furthermore, data from a recent study with single cell sequencing of CMs from mice subjected to chronic pressure overload pinpointed a central role for mitochondrial function and biogenesis in the development of cardiomyocyte hypertrophy and transition to heart failure [82].

There are several approaches to promote mitochondrial health in the aging heart. Elimination and targeting the production of ROS by antioxidants, enhancing mitochondrial DNA repair, and mitochondrial autophagy (mitophagy) have all been suggested to be important mediators of longevity. Interestingly, a number of cellular signaling mechanisms known to extend lifespan also appear to preserve mitochondrial function. SIRT1 is a nicotinamide adenine dinucleotide (NAD+)-dependent deacetylase that plays a key role in longevity and in maintenance of mitochondrial homeostasis [83]. Transcription factor peroxisome proliferator-activated receptor gamma coactivator 1-alpha (PGC1a) appears to be a central regulatory target of SIRT1, and regulates ROS and mitochondrial biogenesis, especially in the muscle [84].

## 6. Mitophagy

For non-dividing cells, the inability to remove damaged organelles and molecules through proliferation amount to an increase in the importance of autophagy to refresh the cellular contents [85] and to limit induction of further damage to the cells [81]. Mitochondrial homeostasis is sustained through membrane fusion and fission [86], which enables its content mixing and selective elimination of compromised mitochondria by mitophagy [87]. The critical role of mitophagy for maintaining CM homeostasis and cardiac function is implicated in both cardiac aging and in pathologic cardiac conditions [8].

Manipulation of mitochondrial fusion protein *fzo* (homolog of mammalian mitofusins (Mfn1/2)) or conserved fission GTPase protein DRP-1 in *C*. *elegans* showed that the AMPK- and dietary restriction -mediated lifespan extension requires both mitochondrial fission and fusion to preserve mitochondrial homeostasis [88]. Interestingly, combined ablation of drp-1 and fzo-1, which leads to a balanced but static mitochondrial homeostasis, extends lifespan in non-stressed animals, but results in inadequate network plasticity to adapt to metabolic stress [88]. Further, in Mfn1/Mfn2/Drp1 cardiomyocyte triple-knockout mice, the static state of the mitochondria temporarily ameliorates cardiomyopathy compared to single fusion- or fission- deficiency [89]. In long-term, the loss of fission and fusion dynamics in cardiomyocytes represses mitophagy, resulting in impaired mitochondrial quality control as well as impaired clearance of senescent mitochondria, eventually leading to heart failure due to a massive accumulation of senescent mitochondria that displaces and disrupts sarcomere structure in cardiomyocytes [89]. Interestingly, the mitochondrial senescence in the triple KO heart is accompanied by a marked reduction in expression of mitochondrial biogenesis factors PGC1α, PGC1β, and PPARγ.

Studies in mice subjected to cardiac pressure overload showed that cardiomyocyte mitophagy is transiently elevated at days 3–7, followed by a reduction in mitophagy eventually resulting in mitochondrial dysfunction [90]. Repressing the mitophagy exacerbates mitochondrial dysfunction and TAC-induced heart failure, whereas injection of a peptide of autophagy inducer (Beclin-1) mitigates mitochondrial dysfunction and TAC-induced heart failure at least partially by restoration of autophagy and mitophagy [90]. In sepsis-induced heart failure, cardiac-specific overexpression of Beclin-1 protected the mitochondria, ameliorated fibrosis, inflammation, and preserved cardiac function [91]. On the other hand, genetic Beclin-1 deficiency resulted in aggravated sepsis-induced cardiac dysfunction [91]. Beclin-1 is a target of miR-30a, and involved in an axis of long non-coding RNA AK088388/miR-30a/Beclin-1 [92]. Interestingly, augmenting miR-30a in vitro inhibited Beclin-1, but attenuated CM damage and autophagy under hypoxia/reperfusion injury [92].

Autophagy receptors NIX/BNIP3L (BCL2/adenovirus E1B 19 kDa interacting protein 3-like) and FUN14 domain containing 1 (FUNDC1) have been shown to contain recognition sites for a hypoxia-responsive microRNA miR-137 [93]. Augmenting miR-137 abolished hypoxia-induced mitophagy without affecting global autophagy, and restoration of NIX and FUNDC1 without miR-137 response elements resulted in restoration of mitophagy under hypoxia [93]. Expression of miR-137-3p, a mature form of miR-137, was shown to be up-regulated in cardiac tissue of MI patients and in rat hearts following I/R injury, and targeting miR-137-3p antagonized hypoxia-induced apoptosis and oxidative stress in H9c2 cells [94]. Interestingly, the authors also identified KLF15, a regulator of lipid and fatty acid metabolic genes in the heart and the muscle and heart [95,96], as target of miR-137-3p [94]. However, the role miR-137 in regulation of mitophagy in cardiomyocytes in vivo remains to be investigated. Overall, there is increasing evidence showing that mitophagy is crucial for maintaining cardiomyocyte health and cardiac function, and treatments that promote mitophagy may provide novel therapeutic strategies for mitigating cardiac aging. Both balance and dynamics of mitochondrial fission and fusion are critical for mitophagy, and the loss of dynamics in mitochondrial network appears to contribute to aging-related fragility.

### 6.1. PGC-1α

PGC-1α and peroxisome proliferator-activated receptor α (PPARα) have powerful impact on mitochondrial function and biogenesis, as well as cardiac health [97]. A number of studies have demonstrated that genetic or pharmacological activation of PGC-1α prevents telomere shortening and aging-related changes in the heart, as well as in the skeletal muscle and the brain. Overexpression of PGC-1α in cardiomyocytes enhances mitochondrial proliferation and regulates a number of genes involved in mitochondrial energy production pathways [98], whereas global knockdown of PGC-1α in mice compromises mitochondrial function and reduces cardiac ATP levels resulting in compromised cardiac contractile response [99]. Most importantly, by 7–8 months of age, PGC-1α deficient mice exhibits frank cardiac dysfunction and LV chamber dilation, indicating a role of PGC1α in regulating aging related cardiac function decline [99].

The role of PGC-1α in cardiac aging is not limited to its regulation of mitochondrial in CM but also in angiogenesis [97,100]. PGC-1α is induced by hypoxia in vitro, which promotes angiogenesis by induction of all VEGF isoforms independent of HIF but through co-operating with ERRα to directly activate VEGF promoter [100]. PGC1α deficiency in mice abolished blood flow recovery after ischemia in skeletal muscle, whereas transgenic ectopic expression of PGC1α induced neovascularization via VEGF [100].

PGC-1α is a target of miR-29 family [101]. In the heart, this regulation contributed to miR-29 knockout-induced mitochondrial abnormality, heart failure and systemic hypertension [102]. Interestingly, PGC-1α haploinsufficiency prolonged lifespan in miR-29 mutant mice and partially rescued cardiac dysfunction [102]. PGC-1α is also functionally regulated by the lncRNA taurine-upregulated gene 1 (Tug1) [103]. Overexpression of Tug1 in diabetic mice rescued the expression of PGC-1α and improved the mitochondrial bioenergetics in podocytes and protected from diabetic nephropathy. Mechanistically, Tug1 binds to an upstream PGC-1α enhancer element to enhance Ppargc1a promoter activity. In addition, a brown adipose tissue–enriched lncRNA, lncBATE10, functions as a decoy for repressor of PGC-1α, CUGBP Elav-Like Family Member 1 (Celf1), thereby protecting PGC-1α mRNA from repression by Celf1 [104]. However, the role and function of lncBATE10 in regulating the PGC-1α expression and fat browning during aging remains to be investigated.

PGC-1α has also been shown to activate peroxisome proliferator-activated receptor δ (PPARδ) regulating fatty acid oxidation [105]. MiR-199a and miR-214, both of which belong to miR-199a ~214 cluster, modulate mitochondrial substrate utilization for energy production, promoting a deleterious switch from fatty acid to glucose at least partially by direct regulation on PPARδ [76]. Upregulated expression of miR-199a and miR-214 are found in TAC operated mice and in heart failure patients, while PPARδ expression is reduced in TAC operated mice, paralleling with the repression of genes involved in fatty acid metabolism [76]. Conditional deletion of PPARδ in mice triggers rapid cardiac dysfunction, while restoration of which by silencing both miR-199a and miR-214 with an RNA based oligonucleotides improves cardiac remodeling and dysfunction, concurrently with restoration mitochondrial fatty acid metabolism [76].

### 6.2. Sirtuins

Sirtuins are a group of NAD-dependent deacetylases of histone [1,2,106] which function interlaced with mitochondria and aging. The deacetylation of histones lead to tightening of nucleosomes and relative inaccessibility of DNA for transcription. Totally, seven members of sirtuins, SIRT1-7, have been identified and participate in wide range of physiological and pathological processes, especially in terms of exercise, dietary restriction and longevity [1,107].

Short hairpin RNA (shRNA) based deletion of mitochondrial SIRT3 and SIRT5 induces cell senescence and mitochondrial dysfunction [80]; in vivo, genetic forced expression of cardiac Sirt1 in mice displays a dose-dependent effect: at low-to-moderate dose, in aged mice it dampens cardiac hypertrophy, fibrosis and improves LV function. However, Sirt1 expressed at high level (12.5-fold) shortens lifespan and induces cardiac hypertrophy, fibrosis, apoptosis and results in LV dysfunction at 6 months of age [108]. In addition, low-to-moderate Sirt1 mitigates paraquat-induced oxidative stress and cardiac damage, possibly through FoxO1a mediated increase of antioxidant; while higher dose of Sirt1 potentiates oxidative stress and interferes mitochondrial function at baseline [108]. Interestingly, Sirt1 is a bona fide target of miR-34a to regulate cell apoptosis [109], and the inhibitory regulation of Sirt1 by miR-34a has been reported to also potentiate cell senescence in vascular smooth muscle cells [110].

Interestingly, Sirt1 antisense lncRNA transcribed from the Sirt1 antisense strand, has been shown to interact with Sirt1 3′UTR and compete with miR-34a to promote Sirt1 translation [111]. In a subsequent study, overexpression of Sirt1 antisense lncRNA was shown to induce cardiomyocyte proliferation, attenuate cardiomyocyte apoptosis, and to preserve cardiac function after MI [112]. However, the relevance of Sirt1 antisense lncRNA in cardiac aging is not known. Sirt1 is also a target of miR-181a [113]. Cardiac-specific targeting entire miR-181 family uncovers a role of miR-181 family in mitochondrial function: inhibition of miR-181 family blunts ROS production, mitochondrial respiration, and alleviates doxorubicin induced ROS injury [114]. However, detailed analysis of family members reveals that miR-181a/b targets PTEN in cytosol, while miR-181c silences mt-COX in the mitochondria. In vivo, miR-181a/b knockout mice exhibited increased infarct size, whereas miR-181c knockout mice shows reduction of infarct size, all of which suggests the influence of miR-181 family on mitochondrial function might lead to serious consequences in response to cardiac injury [114].

It is well known that the angiogenic capacity is compromised in the aging heart [115]. Endothelial cell (EC) specific knockdown of Sirt1 in mice mimics aging-associated decease in microvasculature capacity, whereas EC-specific overexpression of Sirt1 increases skeletal muscle capillary and exercise endurance, and Sirt1 is necessary for exercise-induced neovascularization [107]. Importantly, restoring Sirt1 co-substrate NAD+ by administration of NAD precursor (nicotinamide mononucleotide (NMN)) counteracts age-related decrease of microvasculature and exercise capacity through Sirt1, which may involve Sirt1 mediated repressing of Notch signal [107]. Recently, comparative biology study among 18 rodent species with highly divergent maximum lifespan (MLS, 3–32 years) showed that Sirt6 activity strongly correlates with MLS: species with longevity have more efficient Sirt6, which is largely regulated by five amino acids [116]. A mutual regulation between Sirt6 and miR-122 has been reported [117], which affects liver metabolism and dysregulation of this interplay correlates with hepatocellular carcinoma. It is not known if a similar relationship also occurs in cardiomyocytes.

## 7. Cardiomyocyte Cell Cycle and Reprogramming

As terminally differentiated cells, the regenerative capacity of cardiomyocytes is almost zero in mammalian adults, while loss of cardiomyocytes, either induced by aging itself or secondary to acquired diseases/injures, seems inevitable with aging. The replenishment of healthy CM population is desirable, whereas the continuous contractile function of the heart to maintain blood supply renders the cardiomyocytes unable to undergo substantial cell morphology change to complete cell division [28]. However, there is emerging evidence showing that it may be possible to overcome the natural barrier and resume proliferation of the adult cardiomyocytes, which not only provide another avenue for counteracting the de-generative aging by regeneration, but also solves the issue of loss after injury or during anti-aging therapy. In the last section, we review the recent literature concerning the CM reprogramming and proliferation and the role of non-coding RNAs in governing those events.

An attractive approach to replenish the cardiomyocytes is direct trans-differentiation of aged resident cardiac fibroblasts, which not only removes senescent fibroblasts but also recycles them to provide additional contractile force to the heart. Previously, forced expression of three cardiac transcriptional factors: Gata4, Tbx5, and Hand2 were shown to directly convert resident fibroblast into CM-like cells (iCM) without passing through stem cell stage [118,119]. Similarly, forced expression of Gata4, Tbx5, Hand2 and Mef2c was sufficient to convert moue tail tip fibroblasts to cardiomyocytes and provided benefit in preserving cardiac function after MI [119]. Recently, by screening 8400 molecules, diclofenac, a non-steroidal anti-inflammatory drug (NSAID), was shown to selectively potentiate the Gata4, Mef2c, and Tbx5 -driven trans-differentiation to cardiomyocytes from postnatal or adult fibroblasts, but not from mouse embryonic fibroblasts [120]. Comparing to other reprogramming drivers, diclofenac not only induces cardiac reprogramming by upregulation of CM signature, but also concomitantly suppresses fibroblast and inflammatory signature. Of note, the efficiency of reprogramming in the adult fibroblasts is lower than that in the postnatal fibroblasts, but the addition of miR-133 was sufficient to enhance the reprogramming efficiency in adult fibroblasts to the level of postnatal fibroblasts [120].

Screening of 875 synthetic human miRNA mimics recently identified 96 miRNAs that were able to trigger immature CM (hiPSC-CM) proliferation [121]. Pathway analysis indicated that most of the miRNAs targeted various components of Hippo/YAP signaling pathway, which is known to be involved in regulation of CM proliferation. Additionally, the comparison of miRNA profile in this study to miRNAs previously found promoting rodent CM proliferation shows marginal overlapping, indicating a strong species difference [121]. A subsequent study of 10 miRNAs involved in CM proliferation further confirms that enhancement of nuclear transcriptional activity of YAP is a common requisite for CM proliferation [122]. This event is achieved in part by repression of miRNA targets that inactivate YAP or mediate YAP degradation [122].

Given the strict morphology of cardiomyocytes requisite for contractile function but limiting proliferation [28], another interesting observation obtained is the cardiomyocyte morphology change observed in response to some of the studied miRNAs [122]. Further study revealed that the pro-proliferative miRNAs induce an alteration of the F-actin and G-actin ratio in favoring assembly state, and one common miRNA target (though not in all case of miRNAs) responsible for the network change is Cofilin2 that enhances the F-to G-actin conversion, repression of which by miRNAs induces polymerization of actin filament [122]. Importantly, with this notion, knockdown Cofilin2 by siRNA induces CM proliferation through YAP activation, while preventing actin polymerization with cytochalasin D abolished this effect, further corroborating the critical role of cytoskeleton network (actin polymerization) in CM proliferation [122].

The loss of proliferative capacity of cardiomyocytes in mammals occurs quickly after birth. A study of the postnatal switch in the heart identified increased expression of miR-128 with the transition from neonatal to adult [123]. Heart-specific forced expression of miR-128 dampens CM proliferation, cardiac function and cardiac regeneration, while knockout of miR-128 in heart in vivo prolongs postnatal CM proliferation partially via its directly regulation of SUZ12 [123]. Importantly, ablation of miR-128 in mice potentiates CM proliferation and cardiac regeneration after MI, attenuates MI-induced fibrosis and cardiac dysfunction [123]. Additionally, microarray screen tracking initial phase of CM reprogramming shows robust upregulation of mitosis-related genes. [124]. In vivo delivery of FoxM1, Id1, and Jnk3-shRNAs (FIJs) in mice was shown to be sufficient to promote CM cycle progression [124]. Further, introducing the FIJs cocktail to mice showed benefit in preserving the cardiac function and reducing fibrosis after MI [124].

Recently, long non-coding RNA CPR (cardiomyocyte proliferation regulator) was shown to regulate cardiomyocyte proliferation and cardiac repair [125]. CPR knockout resulted in increased cardiomyocyte proliferation at P14 as well as in the adult heart and, conversely, CPR overexpression reduced cardiomyocyte proliferation in neonatal hearts. CPR knockout mice subjected to MI showed an increase in proliferative markers in cardiomyocytes in MI border zone, increased LV posterior wall thickness and resulted in better preserved LV systolic function as well as smaller scar size following MI.

Most of the studies on CM reprogramming have focused on re-introduction of adult CM back to cell cycle [28], whereas there is relative lack of information on the G0 phase of CMs, at least in the adulthood, that could provide information on mechanisms underlying the CM resistance to proliferation, including changes in mitochondrial dynamics. The characteristics of the post-mitotic cardiomyocytes seem to contradict the definition of cell senescence, which requires withdrawal of a cell from an active cell cycle. It would be therefore crucial to understand when, and how, does a cardiomyocyte transition from a post-mitotic cell to a senescent cell, especially given the role of senescence in normal embryonic development [126,127]. Nonetheless, in the postnatal heart, transition from the hypoxic environment represses CM proliferation via mitochondrial ROS-induced oxidative DNA damage [128]. In the adult mice, a gradual reduction in oxygen tension resulted in reduced ROS and oxidative DNA damage in the CM, and was sufficient to induce the re-entry of adult cardiomyocytes into cell cycle [129], emphasizing the role of mitochondria in governing adult CM proliferation.

## 8. Concluding Remarks

Aging is a highly orchestrated natural process, but it is also a primary risk factor for a variety of diseases, including CVDs [2]. As the demography shift into an older population [1], it is imperative to advance our knowledge of aging to dissect its role from diseases. The co-presence of cardiac aging phenotype with several cardiac pathologies warrant a thorough analysis of their relationship and underlying mechanism. Intervention of cardiac aging by clearance of senescent cells appears attractive, but a more targeted approach warrants further study. The current data on cardiac senescence has mainly focused on non-cardiomyocytes, while characterization of senescent cardiomyocytes is overlooked. Increasing evidence indicates cardiomyocyte mitochondria as the central culprit in aging and promoting/preserving mitochondrial health appears a promising avenue to mitigate cardiac aging. For therapeutic approaches, RNA-based techniques, e.g., shRNA, siRNA and mimics/antagomiRs of miRNAs, may provide higher flexibility to manipulate the mitochondrial health and function. In addition, a number of microproteins produced by lncRNAs and circRNAs have been associated with mitochondria [130], but their role in cardiac aging remains to be investigated.
